# T_H_1-Polarized T_FH_ Cells Delay Naturally-Acquired Immunity to Malaria

**DOI:** 10.3389/fimmu.2019.01096

**Published:** 2019-05-17

**Authors:** Xi Zen Yap, Lucie S. P. Hustin, Robert W. Sauerwein

**Affiliations:** ^1^Department of Medical Microbiology, RadboudUMC Centre for Infectious Diseases, Nijmegen, Netherlands; ^2^Institut Curie, PSL Research University, CNRS UMR168, Paris, France

**Keywords:** T_H_1, T_FH_1, IFN-γ, follicular T helper cells, B cells, malaria, humoral immunity

## Abstract

Humoral immunity is a critical effector arm for protection against malaria but develops only slowly after repeated infections. T cell-mediated regulatory dynamics affect the development of antibody responses to *Plasmodium* parasites. Here, we hypothesize that T follicular helper cell (T_FH_) polarization generated by repeated *Plasmodium* asexual blood-stage infections delays the onset of protective humoral responses. IFN-γ production promotes polarization toward T_FH_1 and increased generation of regulatory follicular helper cells (T_FR_). Delineating the mechanisms that drive T_H_1 polarization will provide clues for appropriate induction of lasting, protective immunity against malaria.

## Naturally-Acquired Immunity in Malaria

Only after years of continued exposure to *Plasmodium* parasites do individuals from malaria endemic regions develop clinical immunity (CI), that protects against clinical disease but not from parasitaemia (1). This protection is mediated through both cellular and humoral immune effector mechanisms. In particular, humoral immunity (HI) apparently plays a pivotal role against blood-stages, which are responsible for pathology and disease. Seminal findings demonstrate that IgG transfer from malaria-immune adults to children with acute malaria can indeed reduce symptoms and parasite load (2).

Effective HI induction requires B cells to be activated by antigen-presenting cells (APCs), predominantly dendritic cells (DCs). Sustained “help” from cognate CD4^+^ T cells is subsequently required for B cell proliferation, affinity maturation, and Ig class-switching. T follicular helper cells (T_FH_), which co-localize with B cells in the germinal centers (GCs), are crucial for both naïve B cell activation during primary infections and reactivation of memory B cells (MBC) in secondary infections. T_FH_ and other CD4^+^ helper T cells (T_H_) can drive naive B cells to differentiate into high-antibody-producing plasma cells (PC) or MBC, which rapidly reactivate and produce specific Abs during secondary infections.

While typically taking a number of years to develop fully, clinical malaria immunity is of relatively short duration and rapidly wanes in the absence of re-infection (3, 4). Antibody efficacy and specific MBC counts increase gradually with age and cumulative exposure, resulting in a strong T_H_1 (IFN-γ-producing) immune response (5–9). The origins of the relatively slow acquisition of clinical immunity, however, remain elusive.

Here we hypothesize that T cell responses generated by repeated blood-stage malaria infection may in fact delay the onset of potent humoral responses. We contextualize the role of T_H_ and T_FH_ polarization surrounding the B cell response in malaria, and suggest that excessive polarization toward the IFN-γ producing T_H_1 phenotype reduces the longevity of antibody responses.

## B-cells and Plasma Cells Are Deregulated in Malaria

Potent humoral responses are characterized by the generation of specific and high-affinity long-lived PCs (LLPCs) and MBCs in the GCs. Yet both adults and children in malaria endemic areas show a delay in the development of MBC and short-lived antibodies targeting *P. falciparum* blood-stage antigens (10). Accordingly, antibodies generated during one acute malaria season are undetectable by the next (10). Similar delays in CI onset are found in malaria-naïve immigrants to Papua New Guinea (11).

Sustained parasitaemia may be a key factor affecting B cell differentiation. Recent studies have provided valuable insights into B cell subset dynamics and antibody kinetics in the context of *Plasmodium* infection. While it is clear that IgG^+^ MBCs are key effectors in long-term memory, high levels of non-IgG^+^ anti-*P. falciparum* MBCs may have a role in early protection (12). Frequent exposure to asexual parasites, as experienced in highly malaria-endemic regions, is associated with the development of MBCs with reduced memory function, known as atypical memory B-cells (AMBC). While the presence of AMBCs may contribute to the delayed and short-lived nature of HI to malaria (13), their presence may also be symptomatic of a more broadly deregulated humoral response.

Frequent parasite exposure seems to be a driving factor in AMBC development. AMBC frequency increases proportionate to transmission intensity, age, and cumulative malaria exposure (13–19), and AMBC proportions increase after each acute malaria episode (20). Conversely, the percentage of AMBCs declines in the absence of parasite exposure, inducing stable populations of malaria-specific classical MBCs (17, 19, 21, 22). This may be the result of direct B cell interactions with *Plasmodium* parasites, or indirectly generated by the pro-inflammatory environment (23, 24), or by a combination of the two, i.e., AMBCs as a product of persistent antigen engagement by B cells within a highly inflammatory environment of chronic malaria exposure, driven by T_H_1 cells (25).

Hence, inappropriate IFN-γ production may be a reflection of inadequate T cell help caused by frequent exposure to blood-stage *P. falciparum*.

## Blood-Stage Infection Induces Changes in T Cell Phenotypes and Populations

Malaria parasites typically induce human T cells with high surface expression of PD-1 and LAG3 and high production of both IFN-γ and IL-10 (26–28). Hence, CD4^+^ T cells in the malarial environment frequently display a phenotype associated with immunosuppression. Furthermore, the malarial environment polarizes CD4^+^ T cells toward the IFN-γ-producing T_H_1-like phenotype, consequently reducing B-cell responses by suppressing antibody-inducing T_H_2 and T_FH_ lineages. While this may be beneficial for containing parasite-mediated pathology, it may contribute to immunopathology and limit reactivation of long-lived MBC. Modeling analyses by Lonnberg et al indicate that monocytes in particular have a role in regulating the T cell response, producing cytokines which skew naïve cells away from the T_FH_ lineage and toward a T_H_1 phenotype (26).

## The Impact of T_FH_ Cells on Humoral Immunity

The T_FH_ subset is particularly crucial for B cell development in the GC and the subsequent generation of a functional memory B cell compartment. T_FH_ responses are widely hypothesized to be disrupted in malaria, as reflected by the relatively high frequency of autoreactive AMBCs and classical MBCs (29).

Due to the challenges of obtaining secondary lymphoid tissue, human research on T_FH_ cells has primarily concentrated on circulatory CD4^+^CXCR5^+^ T_FH_ (30). These circulatory T_FH_ cells share functional characteristics with GC T_FH_ cells including IL-21 production and the ability to induce B cell differentiation *in vitro* (31). They also have properties of a central memory-like T_FH_ population (26, 31–34). In contrast to GC-resident T_FH_, however, circulatory T_FH_ cells lack BCL6 expression, which is required for survival and induction of secondary antibody responses (31, 35–38). BCL6 re-expression can be induced by re-challenge with cognate MBC (39), indicating that sustained antigen presence is required for T_FH_ function.

In the last decade, circulatory T_FH_ subsets equivalent to T_H_1, T_H_2, T_H_17, and T_REG_ have been characterized in mice and humans (40, 41). T_H_1-like T_FH_ cells (T_FH_1) show reduced potential to provide adequate help during antibody maturation *ex vivo* compared to T_H_2-like T_FH_ cells (T_FH_2) (33, 35, 42). The concept that T_FH_ subset imbalance may affect development of antimalarial immunity has gained more traction due to T_FH_ subsets' potential roles in other chronic diseases, such as HIV (43). In parallel, polarization toward T_H_1-like responses has been well-documented in malaria and causes fundamental changes in multiple cell subtypes, such as induction of Th1-like regulatory cells (T_REG_1) (6, 28, 44).

**Figure 1:**
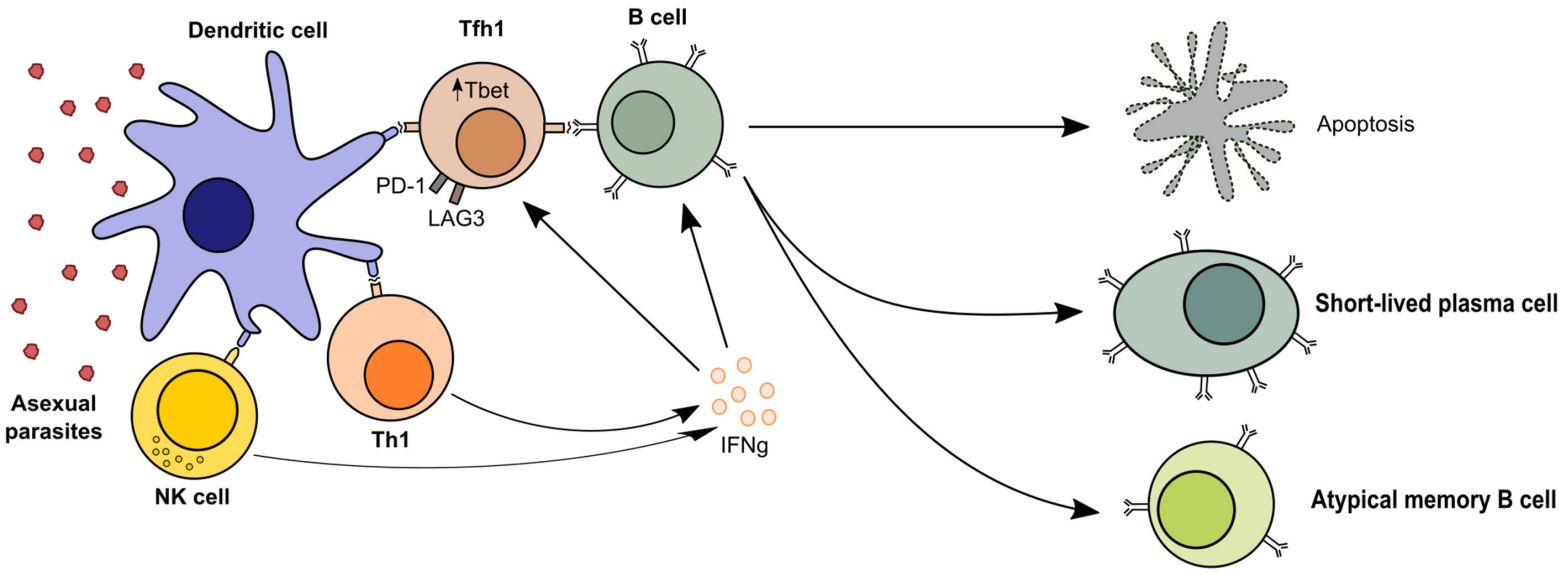
T_H_1-like T cell responses in malaria. Follicular T helper cells are required for B cell activation and the generation of humoral immunity, but malaria profoundly affects T cell polarization and leads to short-lived antibody responses. The presence of asexual parasitaemia promotes activation of T_H_1 and NK cells, which produce high levels of IFN-γ. This microenvironment promotes cellular upregulation of exhaustion markers like LAG3 and PD-1 and T_FH_ differentiation into T_FH_1 cells, which are less effective at activating B cells. Quality of the T cell help in malaria-driven inflammation is therefore reduced, leading to B cell apoptosis or differentiation into short-lived plasma cells and atypical memory B cells, which are poor contributors to the long-term maintenance of humoral immunity.

Thus, dysfunctional GC processes and inappropriate T_FH_ reactions are a likely consequence of malaria infection. Indeed, polarization of T_FH_ is observed in Malian children, with more activated T_FH_1, more T_H_1-like cytokine responses, and less prominent T_H_2 polarization (26, 34, 45–47). This T_H_1-like cytokine response may lead to decreased GC reactions and therefore reduced generation and reactivation of T cell-dependent antibody responses (Figure 1).

Murine data suggest that circulatory T_FH_ may represent pre-T_FH_ generated from partly committed T_FH_ lineage cells rather than mature memory GC-derived T_FH_ cells (45). In murine malaria models, frequency of pre-T_FH_ expressing the T_H_1-associated transcription factor Tbet increases after a single *P. bergei* ANKA infection (46). It will be important to clarify whether malaria-induced circulating T_FH_1 are simply pre-T_FH_ generated in the periphery after a single exposure without entering the GC, and if circulating T_FH_2 therefore represent the mature T_FH_ memory pool. This may explain the differential functionality of these two T_FH_ subtypes in malaria. A proper understanding of the relationship between circulating- and GC T_FH_ will be essential to delineate their particular role in the development of HI.

## How Is the T_H_1-like Signature and T_FH_1-like Polarization Realized?

Studies with transgenic murine *P. yoelii* parasites suggest a positive feedback loop induced by Type I interferon and IL-2; T_H_1 cytokines secreted during *Plasmodium* infection increase CD4^+^ T cell responsiveness by up-regulating Tbet and BLIMP-1 (44, 47). Consequently, CD4^+^ T cells gain an increased predisposition to become T_H_1 cells.

Deregulation of humoral malaria immunity may be the result of an increased T_FH_1:T_FH_2 ratio in combination with the efficacy of the individual responses. Sustained polarization toward a T_FH_1 response after a single infection may affect an individual's ability to respond to subsequent malaria episodes. Frequencies of CXCR3^+^CCR6^−^ T_FH_1s increase transiently but significantly during acute malaria, while CXCR3^−^CCR6^−^ T_FH_2 frequencies decrease long-term in response to multiple malaria parasite exposures (48). In addition, T cell co-receptors may play a role in regulating T_FH_ activation, as shown in *P. yoelli*-infected mice, where activation of OX40 leads to up-regulation of IFN-γ (49), resulting in activation of the inhibitory PD-1 pathway. Consequently, T_FH_ help will shut down, resulting in dysfunctional B cell responses including the generation of AMBCs (25) and decreased parasite clearance due to lower specific IgM and IgG titres (49, 50). Therefore, CXCR3^+^ over-activation may be an important albeit not exclusive factor that limits T cell-dependent antibody responses to *Plasmodium*.

Co-infection with other pathogens can also impact humoral immunity to malaria. Multiple murine studies demonstrated that co-infection with murine Epstein-Barr virus analog MHV68 during *P. yoelii* XNL infection led to very high mortality from symptoms of malaria (51, 52). The latter study indicated that mortality was due to loss of humoral immunity by the MHV68 virus via induction of host IL-10 (52). Host factors involved in parasite sensing can also have a role: humanized mice engineered to express a single MHCII haplotype, HLA-DR4 (0401), had higher rates of parasitaemia and morbidity to *P. yoelii* 17XNL infection than mice engineered to express alternate haplotypes. The loss of parasite control was due to downregulation of humoral immunity by overproliferating T_REG_s (53).

## Other Checkpoint Factors Influencing T Cell Differentiation in Malaria

Regulatory T cell subtypes are likely key modulators of HI. The recently characterized regulatory follicular helper T cell (T_FR_) subset is especially relevant for HI regulation. Contrary to T_R_1, which arise from T_H_1, T_FR_ are a FOXP3^+^ subclass derived directly from T_REG_ which express both BCL-6 and BLIMP-1 (54). Crucially, T_FR_ can directly suppress both T_FH_ and B cells in GC reactions and therefore directly affect GC formation (55–59).

T_FR_ have not yet been studied in the context of malaria, even though their importance is indicated by their key role in controlling antibody production in HIV (60). T_FR_ cell functionality is assumed to be determined by their ratio with T_FH_. As the proportion of T_FR_ increases with age, similarly to T_REG_s (57), we hypothesize that T_FR_ have the potential to play a role in the delayed onset of NAI. Murine studies show that the T_FR_ fraction increases with age while the T_FH_ proportion remains constant (60). T_FR_ may therefore progressively regulate the T_H_1 driven over-activation of DCs, T cells and B-cells.

Conversely, a higher T_FR_:T_FH_ ratio may inhibit T_FH_ activation and proliferation, as suggested by T_FR_-induced downregulation of the proliferation marker Ki67 in T_FH_ cells *in vitro*, dampening T_FH_1 activation (61, 62). However, T_FR_ also downregulate the T_H_2-associated cytokines IL-21 and IL-4 in *in vitro* murine studies, potentially leading to marked defects in GC formation, and B cell affinity maturation (61, 63–65). Changes in the T_FR_:T_FH_ ratio may therefore redirect GC B cells toward becoming extra-follicular MBCs and short-lived PCs, therefore further decreasing generation of long-lived high-affinity antibodies (58, 62).

## Summary, Conclusions, and Outlook

Malaria infection induces T_H_1 polarization characterized by the production of IFN-γ. Overproduction of IFN-γ may be central to poor acquisition of HI by polarizing T_FH_ toward T_FH_1 and causing a positive feedback loop of T_H_1 polarization. It will be crucial to understand the specific parasite components responsible for T_H_1 polarization so that we can better target parasite antigens which catalyze T_H_1 polarization.

Malaria-naïve adults and children from low-transmission regions tend to generate strong pro-inflammatory responses: T_H_1 cytokines IFN-γ and TNFα, and other pro-inflammatory cytokines such as IL-1β and IL-6, are produced, which may favor generation of T_H_1-like responses. However, children with sustained parasitaemia develop a cytokine signature consisting of IFN-γ, Type I IFN, and regulatory cytokines IL-10 and TGF-β (9, 66, 67). It is unclear whether this is related to parasite density, incidence of infections, or both. Parasite burden and transmission intensity could affect T_FH_ polarization through systemic cytokine-mediated effects.

Dendritic cells and NK cells may be responsible for maintaining T_H_1 polarization. Malaria could affect early T cell polarization by disrupting dendritic cell function (68, 69), and DCs co-incubated with blood-stage parasites *in vitro* are shown to polarize naïve T cells toward a T_H_1-like phenotype that produces IFN-γ and TNFα (70, 71). Furthermore, DCs are required for NK cell activation to blood-stage parasites (72). NK cells are major producers of IFN-γ, and rapid reactivation of NK cells in response to blood-stage infection could lead to the formation of a T_H_1 cytokine signature, thereby inhibiting development of positive HI-forming responses. The presence of memory-like responses (trained immunity) from NK cells upon re-encountering pRBCs *in vitro* (73) suggests that NK cell activation in response to malaria may occur rapidly after the first infection, increasing early tendencies toward Th1-like responses. Moreover, NK cell cross-talk with dendritic cells is important for CD4 T cell priming in murine malaria models (74, 75), suggesting that NK cells may bias T_H_1 polarization through multiple pathways.

However, it is unclear whether the blood-derived T_FH_ differ functionally from their GC counterparts. Better models of T_FH_ will be required to study these differences and assess the functional relationship between T_FH_ subsets and the generation of humoral immunity more thoroughly: what phenotypes are generated by B-cells co-stimulated by T_FH_1, the quality of the antibody response, and whether their ability to differentiate into LLPCs or classical MBCs is impacted by malaria-generated T_FH_1s. A culture system to induce T_FH_ or novel systems such as humanized mice which could generate larger quantities of T_FH_ and even allow for isolation of tissue-resident T_FH_ would permit further, in-depth study of these cells. This would also permit mechanistic studies into how T_FH_1 polarization occurs.

In summary, malaria infection, especially repeated infection with high parasitaemia, may generate “inappropriate” T_H_1-like T cell responses that fail to provide the adequate environment for long-lasting HI. This may be due to (i) compromised T_FH_ help, reducing the generation of functional GC and development of typical memory B-cells, leading to a loss of HI longevity; (ii) increased proliferation of regulatory subsets such as T_FR_ which may further inhibit HI by decreasing T_FH_ activation and proliferation; (iii) a strong T_H_1-like immune signature characterized by high production of IFN-γ, illustrated by the increased fraction of T_H_1 and other T_H_1-like cells, including the T_FH_1 subset. To break the cycle, we need improved methods to study T_FH_ and understand the underlying mechanisms of T_H_1 polarization in malaria.

## Author Contributions

XZY and LH wrote the first draft of the manuscript, which was reviewed by RWS. All authors have approved the publication of the final manuscript.

### Conflict of Interest Statement

The authors declare that the research was conducted in the absence of any commercial or financial relationships that could be construed as a potential conflict of interest.
